# Ocular coherence tomography of symptomatic phototoxic retinopathy after cataract surgery: a case report

**DOI:** 10.1186/1752-1947-5-133

**Published:** 2011-04-01

**Authors:** Ahmad M Mansour, Muhammad H Yunis, Walid A Medawar

**Affiliations:** 1 Department of Ophthalmology, American University of Beirut, Beirut, Lebanon; 2 Department of Ophthalmology, Rafic Hariri University Hospital, Beirut, Lebanon; 3 Department of Internal Medicine, American University of Beirut, Beirut, Lebanon

## Abstract

**Introduction:**

High-resolution ocular coherence computed tomography enables unprecedented visualization of the retinal microarchitecture. To the best of our knowledge, this is the first report of high-resolution ocular coherence tomography findings in the healed form of photic post-cataract retinopathy.

**Case presentation:**

A 76-year-old Caucasian man complained of paracentral scotoma, persisting for six weeks after cataract surgery.

**Conclusion:**

Ocular coherence tomography demonstrated a localized juxta-foveal area of retinal atrophy involving the photoreceptor layer, and the retinal pigment epithelium layer.

## Introduction

Operating microscope light-induced foveal damage is a well recognized occurrence following ocular surgery including complicated or lengthy cataract extraction and complex anterior segment procedures [1-5]. While the majority of injuries produce minimal symptoms, scotoma and permanent central vision loss have occurred in some patients. Retinal edema is typically discernable a few days after exposure, while prominent pigmentary changes of the fundus are not apparent prior to two to three weeks after exposure. The recent advent of high-definition ocular coherence computed tomography can help clinicians in analyzing the level and degree of retinal damage after photic damage induced by surgical microscope.

## Case presentation

**Figure 1:**
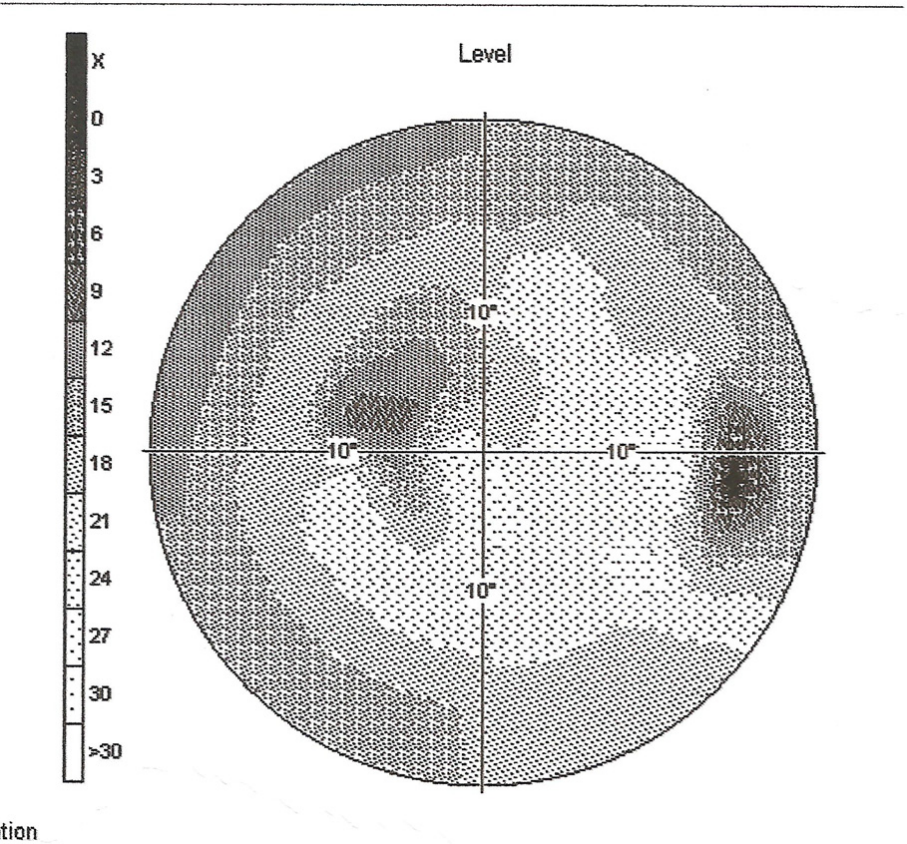
**Automated central 22 field testing of the right eye shows the right blind spot corresponding to the optic disc and the superotemporal paracentral scotoma**.

**Figure 2:**
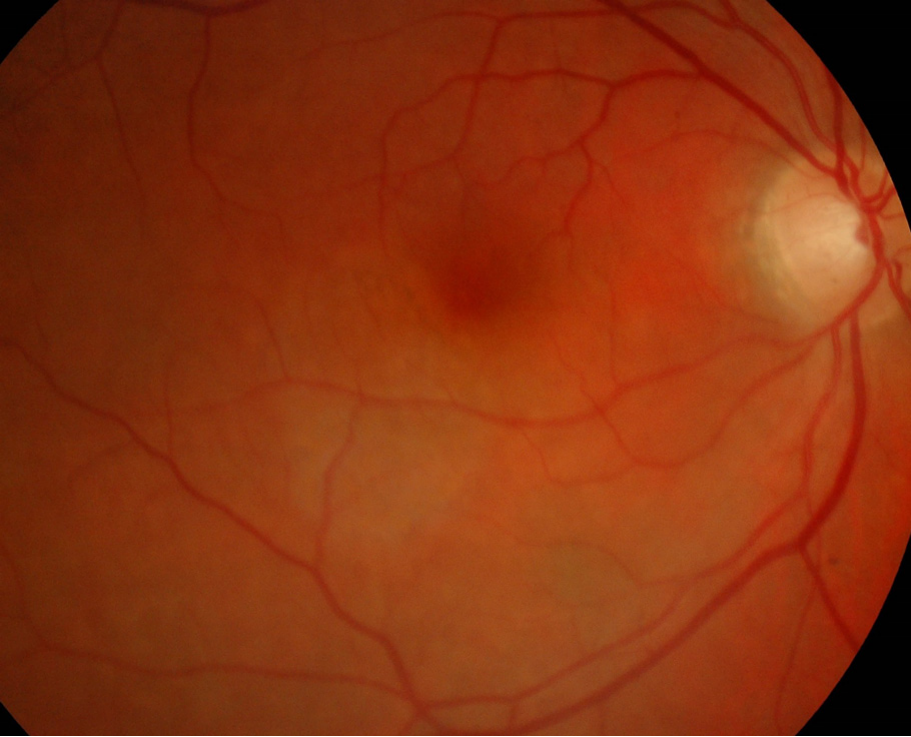
**The fundus photograph shows a well demarcated, elliptical, yellowish, mottled retinal pigment epithelium alteration approximately twice the size of the optic disk, and encroaching on the fovea**.

**Figure 3:**
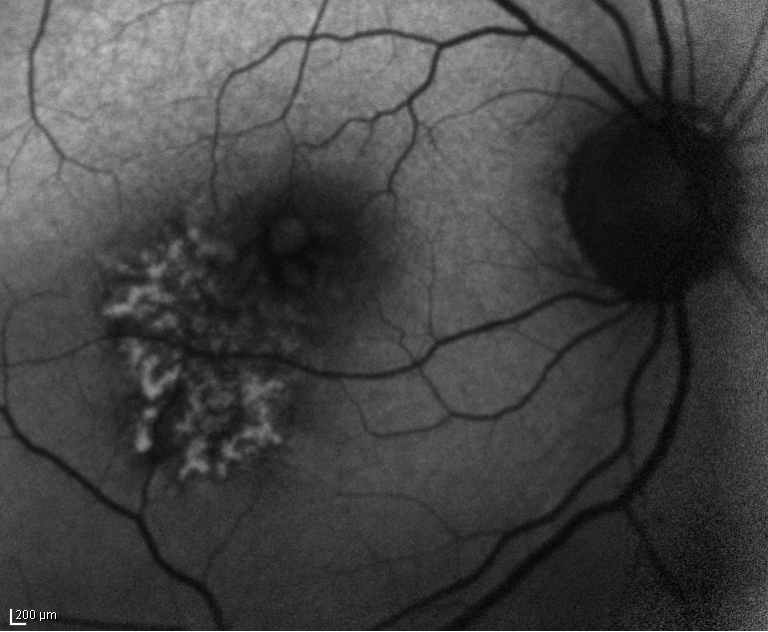
**Autofluorescence image of the posterior pole of the right eye shows a dendritiform pattern of autofluorescence at the inferotemporal macula (BluePeak Blue Laser Autofluorescence, Heidelberg Engineering GmbH, Heidelberg, Germany)**.

**Figure 4:**
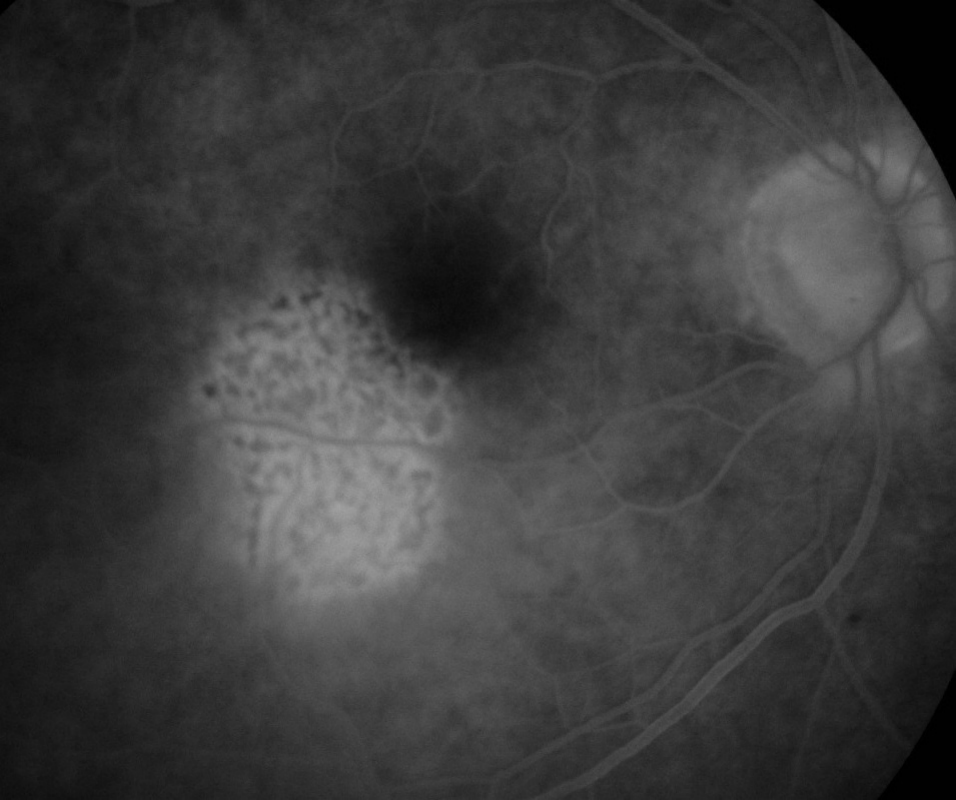
**Fluorescein angiography shows an irregular fluorescein transmission pattern without leakage**.

**Figure 5:**
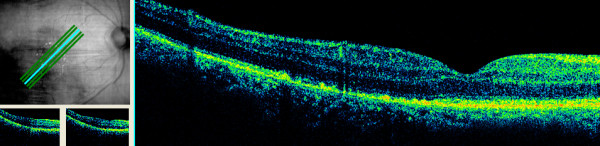
**At six weeks after surgery, a spectral domain ocular coherence tomography (OCT) scan (scan angle 45°, 0.25 mm spacing, 6 mm scan length) showed thinning of the juxta-foveal temporal retinal lesion with effacement of the photoreceptor layer, and retinal pigment epithelium layer (Cirrus, Carl Zeiss Meditec, Oberkochen, Germany)**.

**Figure 6:**
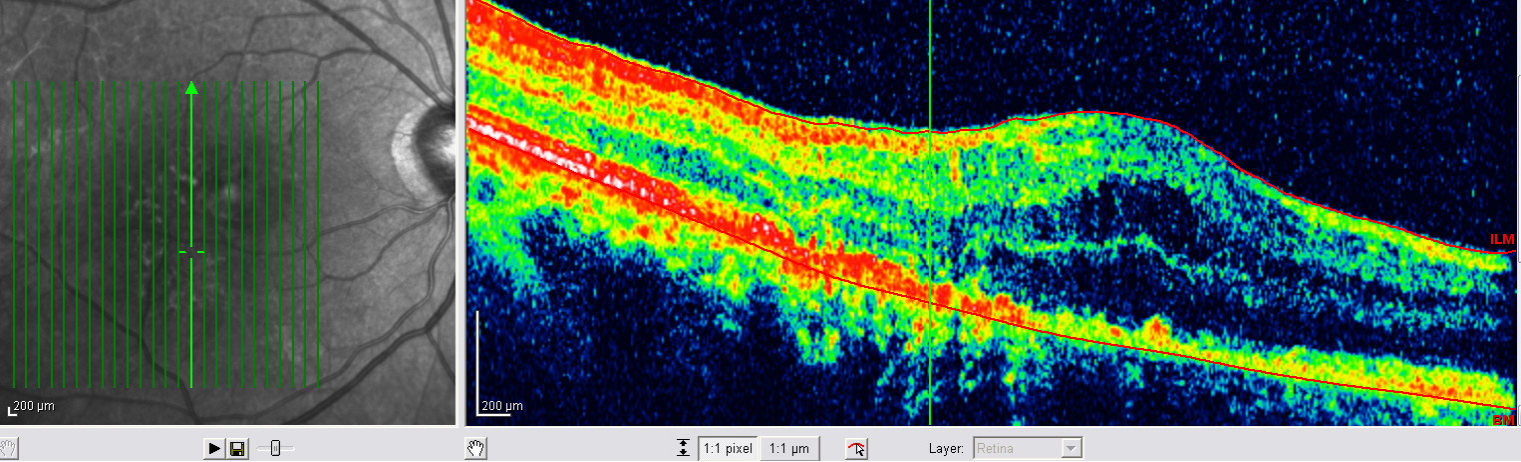
**At nine months after surgery, there is disruption of the photoreceptor layer and retinal pigment epithelium layer with intact Bruch's membrane, as seen on spectral domain ocular coherence tomography (OCT) imaging of the temporal macula (Spectralis, Heidelberg Engineering GmbH, Heidelberg, Germany)**.

A healthy 76-year-old Caucasian man underwent phacoemulsification under retrobulbar anesthesia in his right eye with torn posterior capsule at the completion of cortex aspiration. Anterior vitrectomy was performed and a 5 × 6 mm intra-ocular lens was implanted in the sulcus. A coaxial illuminated microscope (OPMI CS-XY; Zeiss, Oberkochen, Germany) was used for surgery that lasted 45 minutes. At six weeks postoperatively, his best corrected visual acuity was 0.5. His main complaint was a paracentral scotoma confirmed by perimetry (Figure 1). Fundoscopy revealed a well circumscribed flat yellowish retinal lesion, approximately double the disc diameter in size, inferotemporal to the fovea (Figure 2). The retinal lesion was prominent by autofluorescence (Figure 3) stained with fluorescein dye with speckled blockage of fluorescence (Figure 4). Spectral domain ocular coherence tomography (OCT) confirmed thinning of the retinal lesion with loss of the inner/outer photoreceptor layer and retinal pigment epithelium (Figure 5). At nine months after surgery, repeat OCT revealed cystoid macular edema induced by topical travoprost initiated over the past month to control ocular hypertension (Figure 6). There was persistent disruption of the inner/outer photoreceptor layer and retinal pigment epithelium.

## Discussion

Most mild phototoxic retinal injuries probably remain undiagnosed in routine postoperative examination [1-5]. Retinal phototoxic lesions first appear a few days after exposure as well circumscribed outer retinal whitening with mild disturbances of the retinal pigment epithelium, often with a light border. After the first week, lesions are characterized by coarse alterations of the retinal pigment epithelium layer with fluorescein angiography demonstrating sharply demarcated characteristic early discrete mottled hyperfluorescence with late staining. Historically, these lesions are typically located inferior to the fovea as a result of the slight down gaze during extracapsular cataract surgery. The shape of the lesion often matches the shape of the illuminating source of the particular operating microscope used. Such lesions were noted in 3% of the most recent cataract surgery series [4], even in phacoemulsification of short duration. While the majority of injuries produce minimal symptoms, scotoma and permanent central vision loss have occurred in some patients [3,5]. Risk factors for retinal photic injuries have included angle of light incidence, light intensity, exposure time, and intensity of the blue light component [1-5]. It is recommended to use the minimal light intensity needed to perform surgery, use oblique light or filters or pupil shields. Implantation of an intra-ocular lens, including multi-focal lenses, is an important factor in the production of maculopathy [2], on account of its light-focusing effect on the retina.

Acute histological changes in photic injuries have included localized necrosis of the retinal pigment epithelium, extensive disruption of the outer lamellae of the photoreceptors, and edema of the inner segments [1]. Rodriguez-Marco *et al. *[5] presented late OCT findings in a 39-year-old patient who underwent two consecutive pterygium surgeries lasting 1.5 hours. Visual acuity was 0.4 with metamorphopsia. The fundus exhibited a hypo-pigmented rounded lesion in the macular area with early hyperfluorescent foveal area on fluorescein angiography. OCT revealed a detachment of the retinal pigment epithelium.

## Conclusion

We present, to the best of our knowledge, the first report of high-definition OCT findings in the healing stage (six weeks and nine months after surgery) of photic post-cataract retinopathy, showing atrophy of the photoreceptor and retinal pigment epithelium layers.

## Consent

Written informed consent was obtained from the patient for publication of this case report and any accompanying images. A copy of the written consent is available for review by the Editor-in-Chief of this journal.

## Competing interests

The authors declare that they have no competing interests.

## Authors' contributions

AM analyzed and interpreted our patient's fluorescein angiography and OCT data. MY performed the surgery and eye examinations of our patient. WM was a major contributor in writing the manuscript. All authors read and approved the final manuscript.
